# Protocol for the *in vivo* quantification of mitochondria-associated ER membranes in *Caenorhabditis elegans*

**DOI:** 10.1016/j.xpro.2026.104592

**Published:** 2026-05-22

**Authors:** Fivos Borbolis, Konstantinos Palikaras

**Affiliations:** 1 Deparment of Biology, University of Patras, Patras 26504, Greece; 2 Deparment of Physiology, School of Medicine, National and Kapodistrian University of Athens, Athens 11527, Greece

**Keywords:** Cell Biology, microscopy, Model Organisms

## Abstract

Mitochondria-associated membranes (MAMs) are specialized contact sites between the endoplasmic reticulum and mitochondria, with multiple functional aspects. Here, we present a confocal microscopy-based protocol for quantifying the area of MAM-like domains in intestinal cells of the nematode *Caenorhabditis elegans* using organelle-specific fluorescent reporters. We describe steps for worm synchronization, microscope setup, sample preparation, and image acquisition. We then detail procedures for manual single-image analysis and scalable automated batch processing, enabling robust quantification of ER-mitochondria contacts across experimental conditions.

For complete details on the use and execution of this protocol, please refer to Roussos et al.^1^

## Highlights

• Imaging-based protocol for *in vivo* quantification of MAMs in *C. elegans*
• Guidelines for generating synchronous *C. elegans* populations for imaging
• Steps for mounting worms and acquiring confocal fluorescent images of intestinal cells
• Instructions for manual and automated Fiji-based analysis of ER-mitochondria contacts

## Abstract (editor highlights)

Publisher’s note: Undertaking any experimental protocol requires adherence to local institutional guidelines for laboratory safety and ethics.

## Graphical abstract

**Figure:**
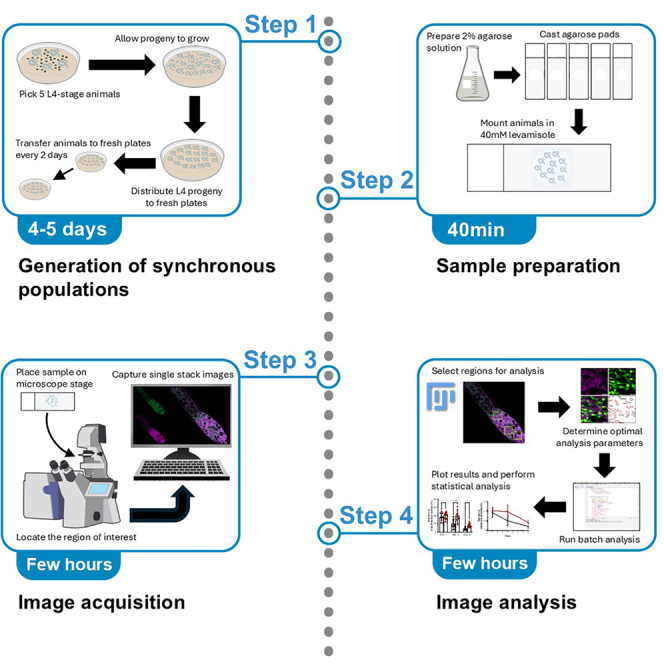

## Before you begin

Functional coupling between the endoplasmic reticulum (ER) and mitochondria is essential for the maintenance of cellular homeostasis, regulating calcium signaling, lipid metabolism and stress responses.^2^^,^^3^^,^^4^^,^^5^^,^^6^^,^^7^ Mitochondria-associated ER membranes (MAMs) constitute key structural features of this interaction, acting as dynamic inter-organelle communication hubs.^8^^,^^9^^,^^10^ As a result, monitoring the behavior of MAMs’ size or structure can provide an important parameter for understanding cellular homeostasis under diverse physiological and/or experimental conditions.

The protocol described below enables precise quantification of the mitochondrial membrane fraction engaged in the formation of MAM-like domains, as determined by their close physical association with the endoplasmic reticulum (ER). It leverages high resolution confocal microscopy combined with a semi-automated image analysis workflow in Fiji (ImageJ), that allows reliable monitoring of changes in the relative area of MAMs during aging or in response to experimental treatments. Although this protocol is optimized for the monitoring of ER-mitochondria appositions in intestinal cells of the nematode *C. elegans* (using the KPA427 strain^1^), it can be adopted for their study in other tissues using the appropriate strains, while our post-imaging analysis pipeline is adaptable and can be readily applied to other experimental systems, including murine or human cells.

### Innovation

This protocol introduces a reproducible, semi-automated workflow for quantifying the fraction of mitochondrial membranes engaged in mitochondria-associated ER membranes (MAMs) in *C. elegans* using high-resolution confocal microscopy and Fiji (ImageJ)-based analysis. Unlike ultrastructural methods such as electron microscopy, which are labor-intensive and low-throughput, or biochemical fractionation, which disrupts native architecture, this approach preserves tissue integrity and enables per-animal, spatially resolved measurements. Moving beyond simple pixel-based colocalization, our workflow quantifies the proportion of mitochondrial surface that is in close physical apposition to ER sites, by specifically analyzing the mitochondrial outline, rather that the full mitochondrial area. This perimeter-based strategy isolates the biologically relevant membrane interface where MAMs are formed and prevents the inclusion of fluorescence originating from different focal planes, ensuring that only true membrane-membrane appositions contribute to the measurement. The method is scalable, easy to implement and adaptable to multiple tissues, strains, or experimental conditions, while offering a physiologically relevant readout, features that are often incompatible with more complex, proximity-based techniques, such as proximity-labeling or split-fluorophores. By combining robust quantification, adaptability, moderate throughput, and broad applicability, this protocol advances existing imaging-based strategies and provides a versatile platform for the systematic study of ER–mitochondria communication and dynamic MAMs behavior in vivo.

### Preparation of *C. elegans* culture media

**Timing: 3 days**

1. Prepare Nematode Growth Medium (NGM) plates – Timing 3 h.
  a. Pour 700mL distilled water (dH_2_O) into a 1L glass bottle or conical flask and add a magnetic stirring bar.
  b. Add 3 g NaCl, 2 g Bacto peptone, 0.2 g Streptomycin and 17 g Agar while stirring on a magnetic plate.
    ***Note:*** Agar powder will not dissolve before sterilization
  c. Fill a separate bottle or flask with ∼400mL dH_2_0.
  d. Cover both containers with their lid or a piece of aluminum foil and autoclave at 121°C for 20min.
    **CRITICAL:** It is important to include a separate bottle/flask of dH_2_O instead of preparing the medium directly in 1L to avoid overflow of the medium during sterilization.
  e. After sterilization, allow the medium to cool while steering to avoid solidification.
    ***Note:*** The medium can be cooled in a 55–60°C water bath, or simply by air until the bottle/flask can be touched for several seconds without causing any discomfort.
    **CRITICAL:** It is essential to allow the medium to cool before adding more ingredients to prevent their precipitation.
  f. Add 1mL CaCl_2_ (1 M), 1mL MgSO_4_ (1 M), 25mL KPO_4_ pH 6 (1 M), 1mL Cholesterol (5mg/mL), 1mL Nystatin (10mg/mL) and use the additional sterilized dH_2_O to fill up to 1 L under aseptic conditions.
    ***Alternatives:*** Instead of using an additional bottle/flask with dH_2_O to use after sterilization, NGM can be directly prepared in a 2L bottle/flask containing 975mL of dH_2_O and the above-mentioned amounts of ingredients.
  g. Pour the medium into 60mm petri dishes (8mL/plate) using a peristaltic pump.
  h. Allow the medium to solidify at for at least 12 h.
    ***Note:*** NGM plates can be stored upside-down at 4°C for up to a month. Avoid storing for longer periods, as evaporation can cause significant alterations in salt concentration.
2. Seed NGM plates with *E. coli* OP50 bacterial food source – Timing 2 days.
  a. Streak a small amount of frozen OP50 from a −80°C glycerol stab in a 90mm LB plate supplemented with streptomycin (200μg/mL) and incubate the plate at 37°C for ∼12 h.
  b. Pick a single bacterial colony and inoculate 50ml of liquid LB medium in a conical flask.
    ***Note:*** OP50 streak plates can be stored at 4°C upside-down and be reused several times for inoculation of liquid cultures. Avoid using streaks older than 10 days to ensure optimal growth and prevent contamination.
  c. Allow the culture to grow at 37°C, while shaking at 200rpm for ∼6 h, to reach an OD_600_ of 0.9–1.2.
    ***Alternatives:*** The liquid OP50 culture can be grown at ∼22–25°C for ∼16h to obtain the same OD_600_.
  d. Apply 200μl of the OP50 culture in the center of each NGM plate under aseptic conditions and gently swirl plates in a cyclic motion to spread bacteria.
    **CRITICAL:** Avoid spreading the culture too close to the edges of the plate to prevent worms from crawling on the wall and drying.
    ***Note:*** Liquid OP50 cultures can be stored at 4°C and be reused for plate seeding. Avoid storing cultures for more than 1–2 days to prevent contamination.
    ***Alternatives:*** Although the use of streptomycin is strongly recommended to prevent contamination and maintain a consistent food source for the nematode, OP50 can be grown in LB and NGM plates without any antibiotics.
  e. Allow bacterial lawn to grow for ∼16 h at ∼22–25°C.
    ***Note:*** Seeded NGM plates can be stored upside down at 4°C for several days. However, it is highly advisable to use freshly seeded plates when performing an experiment.
3. Supplement NGM plates with chemical compounds (optional) – Timing 2 h.
  a. Irradiate seeded NGM plates with 25J/cm^2^ UVC, using a UV crosslinker to render the bacterial lawn metabolically inactive.
    **CRITICAL:** Irradiate plates with their lids off to ensure proper exposure to UV and keep them protected from light after irradiation to prevent the action of repair mechanisms.
  b. Prepare a working solution of each compound in the appropriate concentration so that a volume of 100–200μl will be applied in each NGM plate to achieve the desired final concentration.
  c. Apply the appropriate volume of each compound by spotting with a pipette on the surface of the medium and allow diffusion for 1–2 h before usage.
    **CRITICAL:** Common organic solvents like DMSO and ethanol are toxic for *C. elegans*. For compounds dissolved in these solvents, stock solutions must be prepared in a manner that minimizes the final concentration of solvent in the plate, maintaining levels below 1% for DMSO or 0.5% for ethanol.
    ***Alternatives:*** Irradiation of plates before the application of chemical compounds is highly recommended to ensure that they are not metabolized by the bacterial lawn. However, in cases where compounds are not affected by bacterial metabolism, they can be directly supplemented in the NGM medium after sterilization along with other ingredients (step 1f) or in the OP50 liquid culture before seeding (step 2d).

### *C. elegans* strain maintenance

**Timing: Variable**

4. Maintain a non-starved population of each *C. elegans* strain on OP50-seeded NGM plates at 20°C, by transferring 10 worms into a fresh plate every 3–4 days using a flame-sterilized worm pick (troubleshooting 1).

**CRITICAL:** It is essential to ensure that all strains are well fed for at least 3 generations before starting an experiment. Starvation induces pleiotropic effects on nematode physiology that can interfere with results.

***Alternatives:****C. elegans* strains can also be maintained by cutting a small chunk of medium containing worms of an established population with a flame-sterilized scalpel or spatula and placing it upside-down onto a fresh OP50-seeded NGM plate every 2–3 days. This method allows the bulk transfer of worms, preventing any bottleneck effects that come with picking a small number of individuals, but provides limited control on the life-stage of the animals that are transferred. Additional resources on worm maintenance and handling can be found in the Wormbook chapter “Maintenance of *C. elegans*”.^11^

## Key resources table

**Table:** 

REAGENT or RESOURCE	SOURCE	IDENTIFIER
**Bacterial and virus strains**		

OP50 *E. coli*	Caenorhabditis Genetics Center	OP50-1

**Chemicals, peptides, and recombinant proteins**		

Agar	AppliChem	Cat#A0949
Agarose	Canvax	Cat#AG006
Bacto-peptone	AppliChem	Cat#A2206
Bacto-tryptone	AppliChem	Cat#403682
Calcium chloride dihydrate (CaCl_2_·2H_2_O)	EMSURE	Cat#1.02382
Cholesterol	AppliChem	Cat#A0807
Dimethyl Sulfoxide	AppliChem	Cat#A3672
Dipotassium hydrogen phosphate (K_2_HPO_4_)	AppliChem	Cat#141512
Disodium hydrogen phosphate (Na_2_HPO_4_)	EMSURE	Cat#1.06586
Magnesium sulfate heptahydrate (MgSO_4_·7H_2_O)	EMSURE	Cat#1.05886
Nystatin dihydrate	AppliChem	Cat#A3811
Potassium dihydrogen phosphate (KH_2_PO_4_)	AppliChem	Cat#141509
Sodium chloride (NaCl)	EMSURE	Cat#1.606404
Streptomycin sulfate	AppliChem	Cat#A1852
Levamisole hydrochloride	Sigma-Aldrich	Cat#PHR1798
Urolithin A	Santa Cruz Biotechnology	Cat#SC-475514
Yeast extract	Sigma-Aldrich	Cat#70161

**Experimental models: Organisms/strains**		

*C. elegans*: Stain KPA427: *vkEx2674*[p_*nhx-2*_CemOrange2::PISY-1; p_*myo-2*_GFP];*zcIs17*[p_*ges-1*_mtGFP]	Palikaras Lab	KPA427

**Software and algorithms**		

Fiji (ImageJ)	ImageJ	https://imagej.net/software/fiji/downloads
Excel	Microsoft 365	https://www.office.com
GraphPad Prism	GraphPad Software Inc.	https://www.graphpad.com

**Other**		

Petri dishes Ø60mm	NEST Biotechnology	Cat#754001
Petri dishes Ø90mm	NEST Biotechnology	Cat#752001
Incubator for stable 20°C temperature	POL-EKO	ST 5
Incubator for stable 37°C temperature	Memmert	IN30
Shacking incubator for stable 37°C temperature	Avantor	Cat#444-0550
Dissecting stereomicroscope	Nikon	SZM745
Peristaltic pump	Interscience	Cat#561000
Magnetic stirrer	VWR	Cat#444-0612
Confocal microscope	Zeiss	LSM900
Microscope slides 75mm x 25mm x 1mm	VWR	Cat#631-0701
Microscope coverslips 22mm x 22mm	VWR	Cat#630-2883
Immersion oil	Zeiss	Cat#433802-9010-000
UV crosslinker with 245nm wavelength capability	UVITEC	CL-508

## Materials and equipment

**Table:** KPO_4_ Buffer (1M)

Reagent	Final concentration	Amount
KH_2_PO_4_	0.75 M	108.3 g
K_2_HPO_4_	0.33 M	35.6 g
dH_2_O	-	Up to 1 L
**Total**	**1M**	**1L**

Autoclave and store at ∼22°C–25°C for up to 6 months.

**Table:** NGM

Reagent	Final concentration	Amount
NaCl	50mM	3 g
Bacto-peptone	2.5mg/mL	2.5 g
Streptomycin	0.2mg/mL	0.2 g
Agar	17mg/mL	17 g
CaCl_2_ (1 M)	1mM	1mM
MgSO_4_ (1 M)	1mM	1mL
KPO_4_ (1 M, pH 6)	25mM	25mL
Cholesterol (5mg/mL)	5μg/mL	1mL
Nystatin (10mg/mL)	10μg/mL	1mL
dH_2_O	-	Up to 1 L
**Total**	-	**1L**

Mix NaCl, bacto-peptone, streptomycin and agar in 700mL dH_2_O and autoclave. Cool to 55–60°C and aseptically add CaCl_2_, MgSO_4_, KPO_4_, cholesterol and nystatin from sterile stock solutions. Fill with sterilized dH_2_O up to 1 L and pour into plates (Ø 60mm).

**Table:** M9 buffer

Reagent	Final concentration	Amount
KH_2_PO_4_	22mM	3 g
Na_2_HPO_4_	42.3mM	6 g
NaCl	85.6mM	5 g
MgSO_4_ (1 M)	1mM	1mL
dH_2_O	-	Up to 1 L
**Total**	-	**1L**

Mix KH_2_PO_4_, Na_2_HPO_4_ and NaCl in dH_2_O and autoclave. Cool the solution and add MgSO_4_. Store at RT for up to 2 months or at 4°C for longer periods.

***Alternatives:*** MgSO_4_·7H2O can be added as a powder before sterilization (246,5mg/L).

**Table:** LB medium

Reagent	Final concentration	Amount
NaCl	10mg/mL	10 g
Yeast extract	5mg/mL	5 g
Bacto-tryptone	10mg/mL	10 g
Agar (for plates only)	15mg/mL	15 g
dH_2_O	-	Up to 1 L
**Total**	-	**1L**

Add all ingredients and autoclave. Liquid medium can be distributed to smaller amounts before sterilization. Maintain sterilized liquid LB at ∼22–25°C for up to 3 weeks or at 4°C for longer periods. For LB plates include the agar during preparation and autoclave. Let the medium cool and aseptically add the appropriate antibiotic if needed. For OP50 steaks add Streptomycin at a final concentration of 200μg/mL, using a sterile stock solution. Pour 20mL of LB medium per plate (Ø 90mm). Store plates at 4°C for up to one month.

Stock solutions

• **CaCl**_**2**_ **(1M):** Dissolve 22 g of CaCl_2_·6H_2_O in 100mL dH_2_O and autoclave.
• **MgSO**_**4**_ **(1M):** Dissolve 24.65 g of MgSO_4_·7H_*2*_O in 100mL dH_2_O and autoclave.
• **Cholesterol:** Dissolve 500mg in 100mL of 100% ethanol.
• **Nystatin:** Suspend 1 g in 100mL if 70& ethanol.
• **Streptomycin:** 200mg/mL in sterile dH_2_O.
• **Levamisole stock (0.5M):** Dissolve 1 g levamisole hydrochloride into 10mL of sterile dH_2_O.
• **Levamisole working solution (40mΜ):** Add 80μL of levamisole stock solution to 920μL of M9 buffer.
• **Urolithin A (110mM):** Dissolve 25mg of Urolithin A in 1mL of DMSO.

## Step-by-step method details

### Generation of synchronous nematode populations

**Timing: 4–5 days**

This section describes the preparation of synchronous *C. elegans* populations that will be used for imaging. Animals must be well fed for a minimum of 3 generations before starting any experiment.

1. Pick 5 L4-stage animals in a fresh OP50-seeded NGN plate. Generate 2 plates per strain and condition and incubate them upside-down at 20°C.
2. After 4 days pick 40–50 L4 animals from the progeny and distribute them into 2 fresh OP50-seeded NGM plates (20–25 animals/plate).

**CRITICAL:** Do not place more than 25 animals/plate, as this can lead to overcrowding and starvation when their progeny is generated.

3. Incubate plates upside-down at 20°C. Picked animals will develop into day 1 adults after ∼20 h.
4. Use day 1 animals for imaging, or (if older ages are required) transfer animals by picking into a fresh OP50-seeded NGM plate every second day until they reach the desired life-stage.

**CRITICAL:** When animals of different ages are to be compared, generate multiple synchronous populations, timed so that all required ages can be imaged on the same day. Imaging animals on different days can potentially introduce artifacts due to variations in equipment performance.

***Alternatives:*** Synchronous populations of day 1 animals can also be obtained by allowing 20 gravid adult worms to lay eggs on an OP50-seeded NGM plate for ∼2h and then removing them by picking. Progeny will develop into a synchronous population of day 1 animals after 4 days of incubation at 20°C.

### Casting of agarose pads

**Timing: 30 min**

This section outlines the preparation of thin agarose pads used to immobilize *C. elegans* for high-resolution imaging. These pads provide a stable substrate that minimizes physical stress from the coverslip, thereby preserving worm morphology and enabling extended imaging sessions.

**Figure 1:**
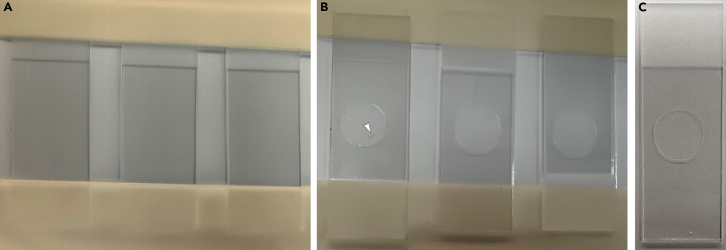
Casting of agarose pads (A) Arrange microscope slides in parallel on the bench and secure two strips of laboratory tape along their outer edges to create a uniform spacer. (B) Dispense molten 2% agarose onto the center of each slide and gently press a second slide on top to spread the agarose into a circular pad. The white arrowhead points to a small bubble. (C) Remove the top slide and allow the pad to air-dry for 5–10 min immediately before use.

5. Prepare a 2% agarose solution.
  a. Add 1 g of agarose to 50mL of M9 buffer in a 100mL conical tube.
    **CRITICAL:** Dissolve agarose in M9 buffer rather than dH_2_O to maintain physiological osmolarity and ionic conditions during imaging.
  b. Heat the mixture in a microwave oven in short bursts until it reaches boiling. Repeat heating cycles as needed, gently swirling the solution between rounds, until the agarose is fully dissolved and the solution becomes clear.
    ***Note:*** Agarose solution can be allowed to solidify and stored at ∼22–25°C for later use after heating. Avoid storing it for more than a month, as evaporation can significantly increase the concentration of agarose and alter pad consistency.
6. Prepare agarose pads.
  a. Arrange microscope slides in parallel on the bench, leaving a small gap between them. Use a number of slides equal to or greater than the number of samples to be imaged.
  b. Apply two strips of laboratory tape along the outer edges of the slides to serve as spacers (Figure 1A).
    ***Note:*** Pad thickness can be adjusted by varying the number of tape layers. Pads that are too thick may distort optical resolution, while overly thin pads dry out quickly and may lead to worm damage.
  c. Place 50-100μL of molten agarose solution onto the center of each slide.
  d. Immediately place a second slide on top, aligned parallel to the first and gently press down at the taped edges to flatten the agarose into a uniform circular pad (Figures 1B and 1C).
    **CRITICAL:** Apply the top slide gently to avoid bubble formation. The presence of small air bubbles (Figure 1B) can be mitigated during sample preparation by positioning worms away from them. However, large or centrally located bubbles may compromise pad integrity and obstruct imaging. Such pads should be discarded and recast.
  e. Allow agarose to dry for a minimum of 10min before use.
    **CRITICAL:** Leave pads covered until shortly before use to protect them from over-drying. Excessive drying of the agarose pad may lead to cracking or reduced transparency.

### Microscope setup

**Timing: ∼1 h**

The following section describes the preparation of a Zeiss LSM900 confocal microscope, equipped with a 40x/1.4 Oil DIC (UV) V-IR immersion objective. While the protocol is tailored to this system, it can be adapted to other confocal platforms with comparable resolution and spectral capabilities. The imaging setup is designed for the *C. elegans* strain KPA427, which co-expresses CemOrange2:PISY-1 under the control of the *nhx-2* promoter, marking the intestinal ER, and a mitochondrial GFP reporter driven by the *ges-1* promoter, labeling intestinal mitochondria. However, it can be adjusted for alternative strains expressing equivalent markers in different tissues.

7. Power on all components of the confocal platform (microscope, laser module, and PC).
8. Launch the Zen software and perform stage calibration.

***Note:*** The protocol was developed using Zen version 3.8.

9. Configure detector parameters:
  a. In the *Acquisition tab*, click on **Smart Setup** to define excitation/emission settings.
  b. Click on +Dyes & Contrast methods.
  c. In the *Dye Database* search for mOrange (or type mOrange in the search box) and add it to the methods list by double-clicking. Repeat for EGFP.
  d. In the *Specific Filter Settings* panel select the **Smartest (line)** option and confirm by clicking **OK.**

***Note:*** Using the “Smart Setup” mode, the system automatically applies the following excitation and emission wavelengths: mOrange, excitation – 561nm, emission – 535-700nm; GFP, excitation – 488nm, emission – 410-535nm.

**CRITICAL:** Imaging settings must be optimized to minimize spectral bleed-through between channels while maintaining acquisition speed sufficient to reduce the possibility of animal movement. Failure to address either issue can result in false-positive signals and imaging artifacts that compromise downstream quantification and interpretation. Although the “Smartest (line)” acquisition mode is well-suited for the separating CemOrange and GFP signals, alternative settings might be more suitable when using different fluorophore combinations.

10. Set acquisition mode parameters:
  a. Scan speed: 3
  b. Direction: bidirectional
  c. Averaging: 4x/Mode: Repeat per Line/Method: Mean Intensity
  d. Bits per Pixel: 8
11. Configure channel-specific parameters (channels submenu):
  a. For the mOran channel (Track1):
    i. Laser line (561nm): 0.8% power.
    ii. Pinhole size: 32μm.
    iii. Master gain: 750 V.
      ***Note:*** For improved visual discrimination between fluorophores during image acquisition and analysis, it is advisable to change the display color of the mOrange channel from orange to magenta.
      ***Note:*** In the imaging setup panel, ensure that the “Switch Track Every” option is set to Line. This setting minimizes temporal offset between channels and reduces the risk of motion-related artifacts caused by small movements of the animals during acquisition.
  b. Select the EGFP channel (Track2) and set the following parameters:
    i. Laser line (488nm): 0.7% power.
    ii. Pinhole size: 1AU (32μm).
    iii. Master gain: 800 V.
      **CRITICAL:** To ensure compatibility with the automated analysis that is described below, the fluorophore labeling the ER must be assigned to channel 1 and the one that marks mitochondria to channel 2. Channel parameters provided here are indicative and should be empirically adjusted to avoid signal saturation while minimizing fluorescence loss. The use of the Range Indicator tool is strongly recommended to guide optimal setup. Once optimized, these settings must remain consistent for all samples within the experiment to ensure accurate quantification.
12. Allow the laser module to warm up for at least 1 h prior to imaging to ensure optimal stability and performance.

### Mounting *C. elegans* for confocal imaging

**Timing: 15–20 min**

The following steps describe the immobilization of *C. elegans* on agarose pads using levamisole, in a manner suitable for high resolution confocal microscopy.

13. Carefully separate the top slide from an agarose pad pair, ensuring the agarose layer remains intact.

**CRITICAL:** Avoid lateral sliding or abrupt lifting, which may tear or wrinkle the pad surface.

14. Allow the exposed agarose pad to dry for a few minutes before preparing the sample.
15. Place a small drop (∼5μL) of 40mM levamisole onto the center of the agarose pad.

***Alternatives:*** Different immobilization approaches (e.g. sodium azide or polystyrene beads) can be used for strains that respond poorly to levamisole or in cases where levamisole affects the biological outcome of the experiment.

16. Mount worms onto the pad.
  a. Use an eyelash tool (eyelash glued to a toothpick) to transfer 10 worms of the desired condition into the drop of levamisole.
  b. Position the worms close together but avoid physical overlap (troubleshooting 2).

***Alternatives:*** Worms can be transferred using a platinum wire pick instead of an eyelash tool. However, this maximizes the risk of injury for animals and introduces excess bacterial lawn in the sample, which can interfere with imaging clarity.

17. Place a 15x15mm coverslip over the pad by a gentle vertical motion to minimize bubble formation and prevent worm displacement.
18. Seal the edges of the coverslip with nail polish to prevent evaporation and protect the sample from immersion oil.

**CRITICAL:** Ensure nail polish is fully dry before placing the slide on the microscope stage to prevent equipment damage.

19. Allow the mounted sample to rest for 5–10min to ensure optimal immobilization of animals prior to imaging.

### Image acquisition

**Timing: Variable (dependent on sample number and imaging depth)**

This section provides a step-by-step guide for the acquisition of confocal images suitable for quantifying the relative area of MAM-like structures in intestinal cells of *C. elegans* using the KPA427 strain in the LSM900 platform. Similar workflows can be applied to alternative strains or adopted to other imaging systems.

20. In the LSM900 platform, select the 40x/1.4 oil immersion objective.
21. Place a small amount of immersion oil to the region of the coverslip directly above the mounted animals.

***Alternatives:*** A small amount of immersion oil may be applied directly to the central surface of the 40×/1.4 oil immersion objective instead of the coverslip. This approach can be used depending on user preference or microscope configuration, provided that optical contact between the objective and sample is maintained.

22. Place the sample slide on the microscope stage, ensuring the coverslip faces downward toward the objective.
23. In the Zen software, navigate to the Locate tab.
24. Use microscope controls to locate the first animal in the sample and navigate to the posterior region of its intestine (troubleshooting 3).

***Note:*** The posterior intestinal region is recommended as the imaging site because it can be consistently identified across individuals, ensuring that the analysis is always performed in the same anatomical domain. This approach minimizes variability arising from intrinsic differences between intestinal cells. Other regions of the intestine can also be examined, provided that the same anatomical segment can be reliably located and imaged in every animal.

**CRITICAL:** To avoid imaging the same animal more than once, implement a consistent navigation strategy across the slide (e.g., scanning from top left to bottom right). Duplicate imaging can introduce bias and compromise data integrity.

25. Activate the Live function in the Zen software to adjust the field of view and briefly monitor fluorescence in both channels.

***Note:*** Animals exhibiting partial or complete transgene silencing may show weak or absent fluorescence on one or both channels. These specimens should be excluded from imaging and downstream analysis to maintain data consistency.

26. Stop Live and use the crop area tool at 2x zoom to isolate a square subregion the corresponds to the end of the animal’s intestine (Figure 2A).

**Figure 2:**
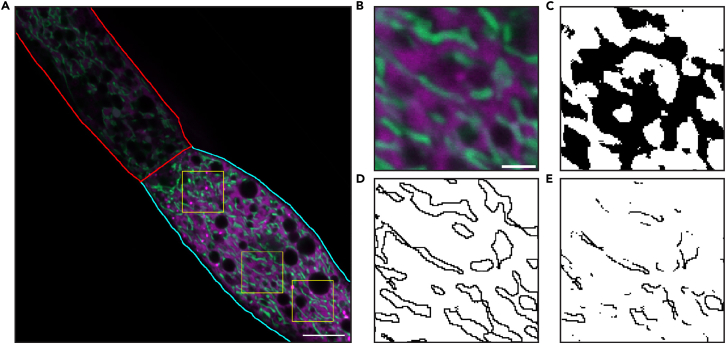
Pre-analysis and single image analysis (A) Selection of regions for sub-image generation. The red outline indicates a poorly focused region that should be excluded from analysis, while the cyan outline marks a well-focused region suitable for quantification. Yellow squares denote representative regions selected for sub-image generation (scale bar: 10μm). (B–E) Expected outcomes of single image analysis. (B) Colocalization image showing merged fluorescence of ER and mitochondria (scale bar: 2μm), (C) binary mask of the ER channel, (D) Mitochondrial outline binary image, (E) resulting MAMs image highlighting regions of physical contact.
27. Reactivate Live and adjust the optimal focal plane using the mouse scroll wheel while holding the Ctrl key to identify the optimal imaging depth.
28. Stop Live, set frame size at 1024 x 1024 pixels and capture the image by clicking the Snap button (troubleshooting 4).

**CRITICAL:** Selecting a higher resolution can improve image quality but will increase acquisition time, raising the risk of motion artifacts due to animal movement. Conversely, using a resolution that is too low may compromise image clarity and produce data unsuitable for quantitative analysis. A resolution of 1024 x 1024 pixels constitutes a good trade-off between image quality and acquisition speed but should be adjusted empirically for each experimental setup.

***Alternatives:*** Z-stack acquisition may be used instead of single focal plane imaging. In this case, downstream analysis can be performed on either maximum intensity projection images or selected single optical sections from each stack. However, analysis on maximum projections should be approached with caution, as even minor internal movements during acquisition can distort spatial relationships and compromise the results. To minimize this risk, a relatively long step size of ∼0.8–1.2μm is suggested.

29. Acquire images from all animals on the slide.

***Note:*** To ensure sufficient sampling and statistical robustness, multiple slides per condition should be imaged. Aim to acquire approximately 15–20 high-quality images per condition, distributed across independent slides to account for technical and biological variability.

30. Save each image as a.czi file (or an equivalent uncompressed format).

**CRITICAL:** All samples must be imaged using identical microscope settings within a given experiment. Variations in acquisition parameters will directly affect fluorescence intensity and may lead to artificial differences in the measured MAMs area. To ensure accurate and comparable measurements, image all experimental conditions in parallel during the same imaging session and maintain stable acquisition parameters across all samples.

### Image pre-analysis

**Timing: Variable (dependent on image number and quality)**

The following steps provide a detailed guide for selecting appropriate regions of interest (ROIs) for quantifying the relative area of MAM-like domains in a single image using the Fiji software.

31. Launch Fiji.
32. Generate a reusable selection pattern:
  a. Create a new image via *File→New* (or press **Ctrl+N**)
  b. Set image type to **8-bit**, dimensions to **125x125 pixels** and click *OK.*
  c. Select the entire image via *Edit→Selection→Select All* (or press **Ctrl+A**)
  d. Add the selection to the ROI manager by pressing **T** and save it using *More→Save* in the ROI manager.

***Note:*** The dimensions of this image define the size of subregions to be analyzed. A size of 125x125 pixels is recommended but can be empirically adjusted. If images were acquired in a 16-bit format, set the new image type accordingly.

33. Open an acquired image in Fiji by drag and drop or via *File→Open.*
34. In the Bio-Formats Import Options pop-up
  a. Set color mode to *Composite.*
  b. Leave all other options unchecked.
  c. Click *OK* to proceed.
35. Select ROIs and generate sub-images for analysis:
  a. Identify a well-focused region with clear signal in both channels and restrict sub-image selection to this region (Figure 2A) (troubleshooting 5).
  b. In the ROI manager, select the saved entry that corresponds to the selection pattern.
    ***Note:*** A saved selection pattern can be reloaded to the ROI manager at any time via *More→Open* or by simple drag and drop of the corresponding file.
  c. Transfer the selection to a representative region of the image (Figure 2A) and duplicate it via *Image→Duplicate* (or press Ctrl+Shift+D).
    **CRITICAL:** In the duplication pop-up, ensure that the “Duplicate hyperstack” is selected to preserve channel information.
  d. Save the duplicated image via *File→Save* (or press **S**)
    **CRITICAL:** Use a consistent and traceable nomenclature that links each sub-image to its original image and experimental condition.
  e. Repeat the duplication process for a second region of the image.
    ***Note:*** For relatively homogeneous images, two subregions are typically sufficient. For more heterogeneous images, select and duplicate three or more regions to ensure representative results in the analysis.
    **CRITICAL:** Manual ROI selection is necessary to ensure that only physiologically relevant regions are analyzed. However, it can introduce user-dependent bias if subregions enriched in or depleted of MAM-like domains are preferentially chosen. This risk can be minimized by selecting multiple ROIs per animal, applying consistent selection criteria and performing the analysis in a blinded manner whenever possible.

### Single-image analysis

**Timing: ∼5 min per sub-image**

This section provides step-by-step instructions for manually quantifying the relative area of MAM-like domains in each sub-image that was generated during pre-analysis using Fiji. While not practical for analyzing large image datasets, this workflow is essential for fine-tuning analysis parameters and should be performed on multiple representative images of each condition to ensure that the appropriate settings have been selected prior to batch processing.

36. Launch Fiji.
37. Open the subimage to be analyzed via *File→Open* (or by drag and drop).
38. Reduce image noise:
  a. Navigate to *Process→Subtract Background.*
  b. In the pop-up window, set **the rolling ball radius** to **50.0 pixels** and leave all other options unchecked.

**CRITICAL:** The optimal rolling ball radius depends on image quality and should be empirically adjusted to reduce background noise without losing information. High-quality images typically require less background subtraction (i.e., higher rolling ball radius), while lower-quality images may benefit from more aggressive subtraction (i.e., lower radius). Use 50.0 pixels as a starting point and enable the **Preview** option on the Subtract Background pop-up to monitor changes in real time. Once selected, this value must remain constant across all images to ensure reproducibility.

**CRITICAL:** The “Light background” option is enabled by default in some distributions of ImageJ. Ensure that this box is **unchecked** before pressing *OK*. Leaving this option checked will result in a uniform “pure color”, effectively destroying the data.

39. Generate a colored duplicate image via *Image→Type→RGB Color* (hereafter referred to as the **colocalization image** (Figure 2B)).
40. Split channels
  a. Select the original image.
  b. Navigate to *Image→Color→Split Channels.*
41. Create binary masks.
  a. Select the mOrange channel image.
  b. Convert it to a binary mask via *Process→Binary→Convert to Mask.*
    ***Note:*** This workflow uses the midpoint of fluorescence intensity in each image as a threshold for generating binary masks. Alternative thresholding algorithms can be used by navigating to *Image→Adjust→Threshold* and selecting the desired algorithm, before converting the image to a binary mask.
  c. Reduce noise via *Process→Binary→Median*, setting the radius to 0.5 pixels (Figure 2C).
  d. Select the GFP channel image and repeat steps 40 b and 40c.
    **CRITICAL:** The radius value for median filtering must be empirically optimized based on image quality for both channels. Once selected, it must remain constant across all images to avoid introducing of artifacts or user bias.
42. Generate mitochondrial outlines.
  a. Select the binary GFP image.
  b. Navigate to *Process→Binary→Outline* to create **mitochondria image** (Figure 2D).
43. Identify the fraction of mitochondrial outlines that colocalizes with the ER (MAMs fraction):
  a. Navigate to *Process→Image Calculator.*
  b. Set Image 1 to the mOrange binary image and Image 2 to the GFP binary image.
  c. Choose the **AND** operation.
  d. Ensure the “Create new window” option is checked.
  e. Click *OK* to generate the **MAMs image** (Figure 2E).
44. Create a selection from the MAMs image via *Edit→Selection→Create Selection.*
45. Validate the accuracy of MAMs identification:
  a. Select the colocalization image (generated in step 38).
  b. Transfer the selection from the MAMs image via *Edit→Selection→Restore Selection* (or press Shift+E).
  c. Confirm that the selection corresponds to the regions around GFP fluorescence that overlap with CemOrange signal.
    ***Note:*** If the LUTs for GFP and CemOrange are set to Green and Magenta, respectively, the selection should mark the grey areas of the RGB image.
  d. If the selection does not successfully represent colocalized pixels, revisit and adjust the rolling ball radius (step 37), the median filter radius (step 40) and lastly the thresholding algorithm (step 41), then repeat the process (troubleshooting 6).
46. Measure mitochondrial surface (MS) area:
  a. Navigate to *Analyze→Set Measurements.*
  b. Ensure **Area** and **Display Label** are selected and leave all other options unchecked.
  c. Select the mitochondria image.
  d. Go to *Edit→Selection→Create Selection.*
  e. Quantify the area via *Analyze→Measure* (or by pressing **M**).
47. Measure MAMs area:
  a. Select the MAMs image.
  b. Go to *Edit→Selection→Create Selection.*
  c. Quantify the via *Analyze→Measure* (or by pressing **M**).
48. Calculate the percentage of mitochondrial surface involved in MAMs by applying the formula $\frac{MAMs\;area}{MS\;area}\times 100$.
49. Calculate the average MAMs fraction across all sub-images derived from a single original image. This value represents the portion of mitochondrial membranes that participate in MAM-like structures for that animal and will be used for downstream plotting and statistical analysis.

### Automated batch-image analysis

**Timing: Variable (dependent on dataset size)**

This section provides the Fiji macro code and detailed instructions for automated batch analysis of large image datasets. The macro replicates all steps of single image analysis to calculate the area of mitochondrial surface (MS) and MAM-like domains across input files. It is specifically designed to process sub-images generated during image pre-analysis and should only be executed after optimal analysis parameters have been selected and validated through single image analysis (see previous section).

50. Launch Fiji and navigate to *Plugins→New→Macro.*
51. Paste the following code:

#@File(label = “Folder", style = “directory") dir

#@File(label = “Save", style = “directory") dir2

#@String(label = “File suffix", value = “.tif") suffix

Dialog.create(“Set Analysis Parameters");

Dialog.addNumber(“Rolling Ball Radius", 50);

Dialog.addNumber(“Median Filter Radius for Channel 1", 0.5);

Dialog.addNumber(“Median Filter Radius for Channel 2", 0.5);

Dialog.show();

rbr = Dialog.getNumber();

C1mr = Dialog.getNumber();

C2mr = Dialog.getNumber();

run(“Set Measurements.", “area display redirect=None decimal=3");

processFolder(dir);

function processFolder(dir) {

 list = getFileList(dir);

 for (i = 0; i < list.length; i++) {

  if(endsWith(list[i], suffix)).

   processFile(dir, dir2, list[i]);

 }

function processFile(dir, dir2, file) {

 setBatchMode(true);

 open(dir + File.separator + file);

 imageTitle = getTitle();

 stop=indexOf(imageTitle, suffix);

 filename=substring(imageTitle, 0, stop);

 run(“Subtract Background.", “rolling=rbr");

 run(“Split Channels");

 C1 = “C1-"+imageTitle;

 C2 = “C2-"+imageTitle;

 selectWindow(C1);

 setOption(“BlackBackground", false);

 run(“Convert to Mask");

 getStatistics(area, mean, min1, max1);

 C1_empty = (max1 == 0 || min1 == max1);

 run(“Median", “radius=C1mr");

 selectWindow(C2);

 run(“Convert to Mask");

 getStatistics(area, mean, min2, max2);

 C2_empty = (max2 == 0 || min2 == max2);

 run(“Median", “radius=C2mr");

 run(“Outline");

 imageCalculator(“AND create", C1, C2);

 C3 = getTitle();

 selectWindow(C2);

 saveAs(“Tiff", dir2 + File.separator + filename + “_MS");

 C4 = getTitle();

 if (C2_empty) {

  run(“Create Selection");

  run(“Measure");

  setResult(“Area", nResults-1, 0);

  updateResults();

  } else {

   run(“Create Selection");

   run(“Measure");

   }

 selectWindow(C3);

 saveAs(“Tiff", dir2 + File.separator + filename + “_MAMs");

 C5 = getTitle();

 C3_empty = (C1_empty || C2_empty);

 if (C3_empty) {

  run(“Create Selection");

  run(“Measure");

  setResult(“Area", nResults-1, 0);

  updateResults();

  } else {

   run(“Create Selection");

   run(“Measure");

   }

 selectWindow(C5);

 if (selectionType() != -1) {

 roiManager(“Add");

 }

 selectWindow(C4);

 if (roiManager(“count") > 0) {

  roiManager(“Select", 0);

  roiManager(“Set Fill Color", “red");

  run(“Flatten");

  saveAs(“Tiff", dir2 + File.separator + filename + “_OVRL");

  }

 roiManager(“reset");

 }

selectWindow(“Results");

saveAs(dir2 + File.separator + “Results.txt");

close(“Results");

**CRITICAL:** Ensure the programming language is set to ImageJ Macro in the *Language* menu of the macro window.

52. Save the macro via *File→Save* in the macro window.

***Note:*** In future uses, the macro can be opened via *File→Open* in the macro window, or by drag and drop.

53. Run the macro and set the appropriate parameters:
  a. Click the *Run* button of the macro window.
  b. On the first pop-up, define:
    i. The path of the input folder that contains the sub-images to be analyzed (Folder).
    ii. The location for saving output files (Save).
    iii. The suffix of the input files (e.g., .tif).
  c. On the second pop-up define the following analysis parameters as determined during single image analysis:
    i. Rolling Ball Radius.
    ii. Median Filter Radius for Channel 1.
    iii. Median Filter Radius for Channel 2.

***Note:*** For each sub-image analyzed, the macro generates three.tif files suitable for data presentation: one depicting the total mitochondrial surface (MS), one showing the fraction that colocalizes with the ER and likely participates in MAMs and one that shows these MAM-like domains as an overlay on the MS. In addition, a.txt file is produced, containing the quantified areas of MS and MAMs for each sub-image (troubleshooting 7, 8 and 9). Further information on data analysis and presentation is provided in the expected outcomes section.

## Expected outcomes

The automated batch image analysis workflow generates three.tif output images for each sub-image analyzed: the first depicts the outlines of the total mitochondrial surface (_MS suffix); the second shows the fraction of mitochondrial surface apposed to the ER that likely corresponds to MAMs (_MAMs suffix); the third is an overlay image combining MAMs and mitochondrial outlines (_OVRL suffix).

**Figure 3:**
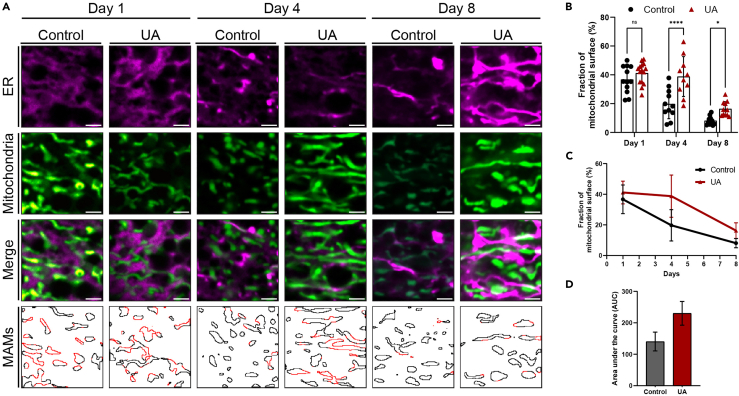
Urolithin A mitigates age-dependent decline in MAMs area (A) Representative image showing ER-localized CemOrange (magenta) and mitochondria-targeted GFP (green) in intestinal cells of animals under control conditions or treated with UA. Panels include single channel fluorescence, colocalization and MAMs overlay images, where black lines depict mitochondrial outlines and red parts correspond to MAMs (scale bars: 2μm). (B–D) Quantification of MAMs area. (B) Bar chart showing average MAMs fraction per animal; mean ± SD; ∗p < 0.05, ∗∗p < 0.01 ∗∗∗P < 0.0001; two-way ANOVA; each point represents one animal. (C) Time course XY scatter plot showing average MAMs fraction across timepoints for each condition. (D) Area under the curve (AUC) values used for integrated quantification.

In addition, the workflow calculates the quantified area (expressed in pixels) for both MS and MAM-like domains. These values are automatically saved into a single.txt file (Results.txt file), where each entry includes the filename label (ending in “_MS” or “_MAMs”) and the corresponding area measurement. Representative output images can be used to generate figures that visualize MAMs area and distribution. For each experimental condition, we recommend including a set of four images: ER fluorescence, mitochondria fluorescence, merged fluorescent channels and the overlay output image (Figure 3A).

In our pilot experiment we investigated the effect of aging on MAMs area under control conditions, or during treatment with Urolithin A (UA), a gut microbiome-derived metabolite of ellagitannins and ellagic acid that is known to induce mitophagy and extend lifespan. Consistent with published literature, our workflow revealed a reduction in MAMs area during aging, validating the robustness of the methodology (Figure 3). Moreover, the application of this method uncovered a previously unreported role for UA in the preservation of MAMs during aging. These findings underscore the reliability of our imaging-based approach for quantifying MAMs and its applicability to aging-related studies and pharmacological interventions. Our experimental conditions can be used as controls in future applications of this protocol. Overall, the workflow can be readily adapted to other experimental models or organelle contact sites, offering a scalable approach for the investigation of inter-organelle communication.

## Quantification and statistical analysis

The data in Results.txt file can be directly imported into spreadsheet software to calculate the percentage of mitochondrial surface involved in MAM-like structures, by applying the formula $\frac{MAMs\;area}{MS\;area}\times 100$. Once calculated for each sub-image, these values can be averaged across sub-images stemming from a single original image (i.e., one animal) and be used for plotting and statistical analysis. Data visualization and analysis can follow two complementary approaches:

1. Time point-specific comparison:

For each condition and time point, plot the average MAMs fraction per animal in a grouped table. Statistical significance can be assessed using a two-way ANOVA. We recommend bar or box plots that display individual animal values as points, with standard deviation (SD) error bars (Figure 3B).

2. Time course analysis with AUC:

For each condition, plot the mean MAMs fraction across time points in an XY scatter plot with SD error bars. Assign X values to time points (e.g., days) and Y values to MAMs fractions. To quantify the overall behavior, calculate the area under the curve (AUC) for each condition (Figures 3C and 3D). AUC This provides an integrated measure of mitochondrial participation in MAMs formation over time, with higher values reflecting greater engagement. This approach enables robust comparison between treatments and can capture temporal trends that may be missed in pointwise analyses. AUC values from individual biological replicates can be used for downstream statistical testing with t-tests or one-way ANOVA.

The first approach offers detailed resolution but is limited to pairwise comparisons. The second provides a holistic view of condition-specific trajectories and is particularly useful when integrating data from multiple biological replicates (expressed as different AUC values).

## Limitations

While this protocol provides a robust and reproducible framework for quantifying MAMs in *C. elegans* intestinal cells, some limitations should be considered. It is important to note that the proposed hardware prioritizes sample throughput and ease of handling over optical resolution, offering a practical alternative to more precise, but labor-intensive setups. As a trade-off, its lateral resolution exceeds the physical dimensions of MAMs, introducing a degree of uncertainty in the measurements. When feasible, key findings should be validated using higher-resolution approaches, such as electron microscopy. Furthermore, the reliability and reproducibility of the workflow is highly dependent on imaging consistency, as fluctuations in microscope performance or acquisition settings across imaging sessions can introduce unwanted variability and technical artifacts. As a mitigation strategy all samples should be imaged in parallel during the same session, even when comparing animals of different ages, and microscope settings must be stable between replicate experiments that take place on different days. Stable transgene expression is also critical, since batch processing applies adjustable but fixed parameters that do not dynamically adjust to variations in signal quality among images. The use of integrated or otherwise stable expression lines is highly recommended, since it substantially reduces variations in fluorescence intensity between cells and animals.

Moreover, manual ROI selection must be carefully performed as it can introduce bias, particularly in heterogeneous samples. The choice of immobilizing agent should also be considered: although levamisole is generally regarded as effective and neutral, its impact on organelle morphology may vary in sensitive strains. Alternative immobilization methods should be considered when working with particularly sensitive animals or experimental conditions. Finally, despite its overall effectiveness, the analysis pipeline relies on 2D projections and thus does not capture the full three-dimensional topology or dynamic behavior of ER-mitochondria interactions, which may limit interpretation in thicker or highly motile regions. Nonetheless, the protocol offers a practical and scalable solution for investigating MAMs dynamics in vivo and can serve as a foundation for further refinement or adaptation to more complex imaging modalities.

## Troubleshooting

### Problem 1

Worms starve during maintenance (related to before you begin – step 4).

### Potential solution

• Ensure that the bacterial lawn is well established by seeding NGM plates with 200μL of fresh OP50 culture (see before you begin – Preparation of NGM plates section).
• Avoid transferring more than 10 adult worms per plate or refrain from transferring large agar chunks.
• Monitor worm populations daily and adjust transfer frequency to fresh plates as needed to prevent starvation.

### Problem 2

Worms overlap or drift during mounting (related to step-by-step method details step 16 b). This issue typically arises from excess liquid during sample preparation that allows worms to scatter.

### Potential solution

• Reduce the volume of agarose to generate smaller pads.
• Limit levamisole volume on pads to 3-5μL.
• Position worms carefully and place the coverslip promptly to minimize drift.

### Problem 3

High number of worms burst during imaging (related to step-by-step method details – step 24). This my result from improper agarose pad preparation (see before you begin – Casting of agarose pads) or mounting technique (see step-by-step method details – Mounting *C. elegans* for confocal imaging).

### Potential solution

• Prepare a fresh 2% agarose solution. Reused or aged solutions may concentrate and reduce buffering capacity, increasing the risk of bursting.
• Increase the volume of levamisole to reduce mechanical pressure on animals.
• Use freshly prepared levamisole solution to ensure correct concentration for both anesthetic and M9 ingredients.
• Minimize imaging time per sample to avoid pad desiccation. If necessary, prepare more slides with fewer animals to keep imaging within 15-20min, while maintaining adequate image yield.

### Problem 4

Imaging artifacts occur due to motion blur or excessive bacterial debris (related to step-by-step method details – step 28).

### Potential solution

To prevent motion blur:

• Increase levamisole concentration to 80mM or higher. This should be done with caution as high levels of anesthetics may affect organelle function or morphology and interfere with results.
• Increase scan speed by adjusting the acquisition settings of the microscope, noting that this may reduce image resolution.
• Use a larger zoom factor, to scan a smaller section and therefore reduce imaging time. Note that this approach may require a higher number of images per animal to obtain representative results.

To reduce bacterial debris:

• Use an eyelash tool instead of a platinum pick for mounting.
• Rinse the eyelash tool in dH_2_O between animals.
• Transfer animals through a small drop (∼5μL) of levamisole before placing them on the pad to wash away excess bacteria.

### Problem 5

Weak or absent fluorescence in one channel (related to step-by-step method details – step 35). This may result from poor acquisition setup, photobleaching or transgene silencing.

### Potential solution

• Increase laser power or gain to improve signal detection during acquisition.
• Minimize exposure during Live mode and use the lowest possible laser power to avoid photobleaching.
• Exclude animals with poor signal to mitigate the impact of transgene silencing. This may require imaging more animals to reach the desired sample size, while meeting qualitative criteria.

### Problem 6

Colocalization selection does not match visual overlap (related to step-by-step method details – step 45). This discrepancy may stem from suboptimal imaging or analysis parameters.

### Potential solution

• Adjust that laser power and gain to capture a robust signal in both channels while minimizing saturated pixels.
• Revisit and empirically adjust preprocessing parameters (rolling ball radius, median radius and possibly the thresholding algorithm – see steps 38 and 41).

### Problem 7

Macro generates no results or fails to process all files (related to step-by-step method details – steps 51–53).

### Potential solution

• Ensure the “file suffix” parameter matches the actual suffix of input files.
• Organize sub-images for each condition into separate folders containing only the relevant files and use these folders as input.

### Problem 8

Macro output images are blank (related to step-by-step method details – steps 51-53).

### Potential solution

• Confirm that ER is assigned to channel 1 and mitochondria to channel 2 in all images.
• Revalidate macro parameters by reperforming single image analysis (see steps 36–49).

### Problem 9

Macro output images cannot be traced to their corresponding sub-image (related to step-by-step method details – steps 51–53). This issue may arise from ambiguous or repeated filenames during acquisition or sub-image generation.

### Potential solution

• Use a standardized file name convention that encodes key metadata (e.g., age and treatment) on the original filename, along with a unique counter during acquisition.
• Preserve original image identifiers when naming sub-images and append a clear ROI index to maintain traceability.

## Resource availability

### Lead contact

Further information and requests for resources and reagents should be directed to and will be fulfilled by the lead contact, Prof. Konstantinos Palikaras (palikarask@med.uoa.gr).

### Technical contact

Technical questions on executing this protocol should be directed to and will be answered by the technical contact, Prof. Fivos Borbolis (fborbolis@upatras.gr).

### Materials availability

All materials are either commercially available or available upon request.

### Data and code availability

Data shown in Figure 3 are available upon request. The published article includes all code generated and used for analysis during this study.

## Acknowledgments

K.P. is supported by grants from the 10.13039/501100000781European Research Council (grant no. ERC-101077374 SynaptoMitophagy); the Fondation Santé; and the Greece 2.0, National Recovery and Resilience Plan Flagship program TAEDR-0535850. The funders had no role in study design, data collection and analysis, the decision to publish, or preparation of the manuscript.

## Author contributions

F.B. conceptualized the protocol, conducted the experiments, performed the analysis, and wrote the manuscript. K.P. supervised the protocol, edited the manuscript, and provided resources and funding.

## Declaration of interests

The authors declare no competing interests.
